# Adaptive immunity against gut microbiota enhances apoE-mediated immune regulation and reduces atherosclerosis and western-diet-related inflammation

**DOI:** 10.1038/srep29353

**Published:** 2016-07-07

**Authors:** Diego Saita, Roberto Ferrarese, Chiara Foglieni, Antonio Esposito, Tamara Canu, Laura Perani, Elisa Rita Ceresola, Laura Visconti, Roberto Burioni, Massimo Clementi, Filippo Canducci

**Affiliations:** 1 Microbiology and Virology Laboratory, San Raffaele Scientific Institute IRCCS, Milan, Italy; 2 Cardiovascular Research Area, San Raffaele Scientific Institute IRCCS, Milan, Italy; 3 Department of Radiology, San Raffaele Scientific Institute IRCCS, Milan, Italy; 4 Centro Imaging Sperimentale (CIS), San Raffaele Scientific Institute IRCCS, Milan, Italy; 5 Department of Biotechnology and Life Sciences, Insubria University, Varese, Italy; 6 Faculty of Medicine, Vita-Salute San Raffaele University, Milan, Italy

## Abstract

Common features of immune-metabolic and inflammatory diseases such as metabolic syndrome, diabetes, obesity and cardiovascular diseases are an altered gut microbiota composition and a systemic pro-inflammatory state. We demonstrate that active immunization against the outer membrane protein of bacteria present in the gut enhances local and systemic immune control via apoE-mediated immune-modulation. Reduction of western-diet-associated inflammation was obtained for more than eighteen weeks after immunization. Immunized mice had reduced serum cytokine levels, reduced insulin and fasting glucose concentrations; and gene expression in both liver and visceral adipose tissue confirmed a reduced inflammatory steady-state after immunization. Moreover, both gut and atherosclerotic plaques of immunized mice showed reduced inflammatory cells and an increased M2 macrophage fraction. These results suggest that adaptive responses directed against microbes present in our microbiota have systemic beneficial consequences and demonstrate the key role of apoE in this mechanism that could be exploited to treat immune-metabolic diseases.

The gut microbiota (GM) has coevolved with humans and, as a multicellular organ, communicates with the host and modulates its physiology^1^. Recently, our group and others demonstrated that the B cells present in both coronary and carotid plaques of patients with cardiovascular diseases locally produce antibodies able to react against GM antigens and to cross-react with self-antigens. We demonstrated that IgG1 immunoglobulins are secreted in human coronary atherosclerotic lesions and recognize the outer membrane proteins of Enterobacteriaceae, such as Klebsiella and Proteus strains, bacteria present in the GM of healthy subjects. Interestingly, this family of bacteria was recently shown to be also a major component of human atherosclerotic lesions-associated microbiome^2^.

The nasal vaccination of hyperlipidemic apolipoprotein E (apoE)-deficient mice with the outer membrane protein of *Porphyromonas gingivalis (P. gingivalis*), a gram-negative microorganism that resides in the oral cavity, reduced atherosclerotic plaques and lowered circulating levels of inflammatory cytokines upon bacterial challenge^3^. The underlying mechanisms are still not fully elucidated, and immunization with bacterial antigens produced significant effects only if atherosclerosis was accelerated through intraperitoneal bacterial inoculation. Moreover, those results were obtained in apoE-deficient mice, a pre-clinical model of atherosclerotic disease and not on wild type animals.

Several genetic risk markers for immune-metabolic diseases such as atherosclerosis, including apoE, are associated with pathogen recognition or inflammatory pathways. In fact apoE, the major component of Very Low Density Lipoproteins (VLDL) is known to modulate plasma lipoprotein clearance, to affect immune activation on antigen presenting cells (i.e. suppression of pro-inflammatory signaling in macrophages and down-regulation of T helper 1-related immune responses)^4^,^5^,^6^, and to be a recognized determinant of cardiovascular disease susceptibility^7^.

Herein, in order to evaluate the effect of systemic immunity against the outer membrane proteins of *Enterobacteriaceae* on atherosclerosis progression and on the proinflammatory status associated with hyperlipidic diet (WD), we immunized C57BL/6 wild type and ApoE^−/−^ mice fed with WD against the outer membrane protein K36 (ompK36) of *Klebsiella pneumoniae*.

We demonstrate that immunized C57BL/6 wild type mice had reduced serum cytokine levels, reduced insulin and fasting glucose concentrations, and both gut and atherosclerotic plaques of immunized mice showed reduced inflammatory cells and an increased fraction of alternatively-activated macrophages. We also demonstrate that the reduced systemic pro-inflammatory state associated with immunization is due to the enhancement of ApoE-mediated immune regulation. Our data clarify that the interplay between genetic background, GM, and acquired immunity: i) is biologically relevant; ii) affects hormone levels, cytokine milieu, pro/anti-inflammatory balance in peripheral organs and atherosclerotic plaque composition also for a long time after immunization; iii) could be therapeutically exploited to protect from WD-associated inflammation and metabolic syndrome.

## Results

### Immunization with ompK36 affects apoE, cytokine and hormone production in serum and gene expression in the liver and visceral adipose tissues

In mice immunized with ompK36 antigen, the titer of circulating antigen-specific IgG antibodies at euthanization was higher than 7 × 10^4^, while no significant (<10) specific reactivity was present in non-immunized control mice (Fig. S1c). Differences in Total Cholesterol (TC), LDL-cholesterol (LDL-c) and Triglycerides (TG) concentrations were not observed between immunized or control animals (Fig. S2a). No significant changes in weight gain or in food consumption were observed between immunized and mock-immunized groups in C57BL/6 or ApoE^−/−^ mice (Fig. S2b,c).

**Figure 1:**
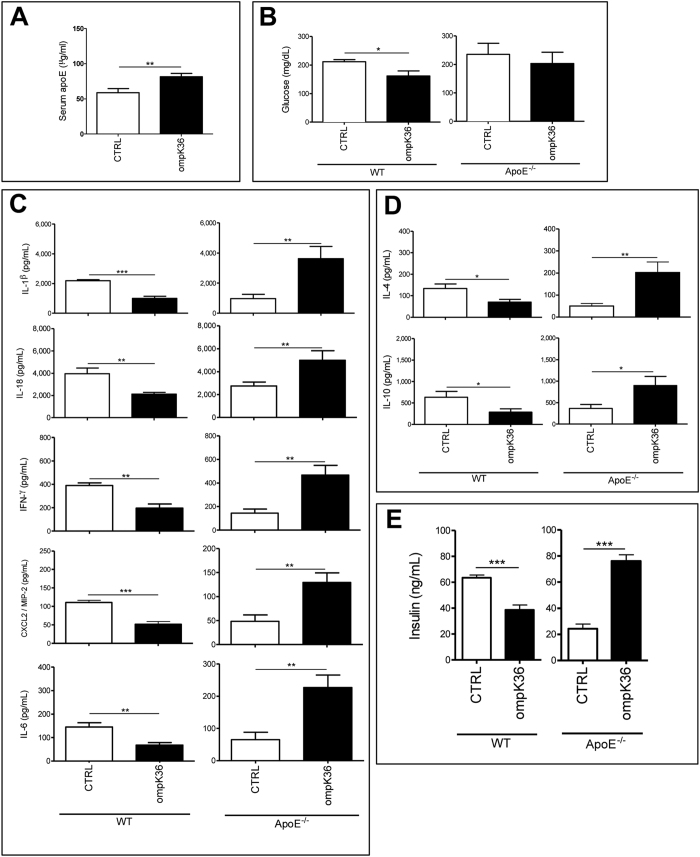
Blood content of inflammatory mediators and hormones in ompK36- and mock-immunized mice fed with WD. Serum levels of cytokines, chemokines and hormones were measured at sacrifice. (**A**) serum level of apoE protein in C57BL/6J mice. (**B**) Glucose, (**C**) selected pro-inflammatory cytokines and chemokines, (**D**) selected anti-inflammatory cytokines and (**E**) hormone concentrations in ApoE^−/−^ and C57BL/6J mice. Statistical analysis was performed by student t test. *P < 0.05; **P < 0.01; ***P < 0.001. Data are plotted as mean ± s.e.m. (n = 7/group).

OmpK36 immunization increased serum levels of apoE protein in C57BL/6 animals fed with WD (Fig. 1A). Interestingly, no changes in the expression level of apoE mRNA could be demonstrated in the liver of ompK36-immunized C57BL/6 mice, even if an increase in plasma levels of apoE protein was confirmed, suggesting that modification of plasmatic apoE was not due to hepatic upregulation.

Significant changes in plasma cytokines and hormone levels were measured in ompK36-immunized mice compared to mock-immunized mice either C57BL/6 or ApoE^−/−^ (Fig. 1C–E and Fig. S3 and S4).

OmpK36 immunization in C57BL/6 mice resulted in a significant reduction of several circulating pro-inflammatory cytokines and chemokines such as IL-1α, IL-1β, IL-18, IFN-γ, IL-6, CXCL2 (Fig. 1C and Fig. S3), IL-5, IL-15 (Fig. S3).

**Figure 2:**
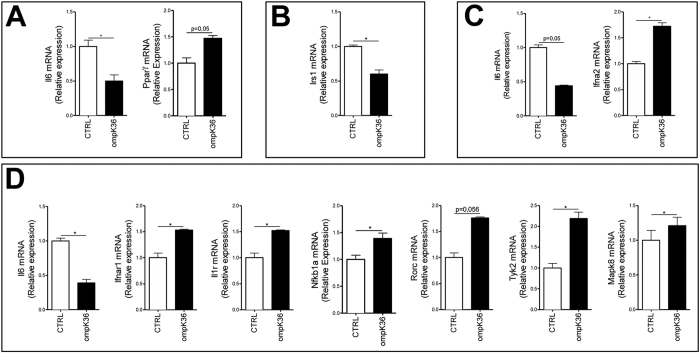
Analysis of gene expression in liver and adipose tissue. (**A**) Liver tissue genes modified by ompK36 immunization in C57BL/6 mice and (**B**) in ApoE^−/−^ mice. (**C**) Visceral fat genes modified by ompK36 immunization in C57BL/6 mice and (**D**) in ApoE^−/−^ mice. *P < 0.05, (n = 7/group).

In C57BL/6 mice, immunization with ompK36 was associated with the following changes in gene expression: in the liver, PPARγ (1,46, p = 0.05) was increased and IL-6 gene expression (−2,02, p = 0.04) (Fig. 2A) was reduced; while in the abdominal adipose tissue, (Fig. 2C) IL-6 (−2,15; p = 0.05) was decreased and IFN-α2 (1,72, p = 0.04) was increased compared to control mice. No significant differences in the gene expression profiles of the spleen, PBMCs, or striated muscles were observed as a result of immunization.

Interestingly, ompK36 immunization decreased fasting glucose and the plasma insulin, level in C57BL/6 mice compared to mock-immunized mice (Fig. 1B,E).

In ompK36-immunized ApoE^−/−^ mice, not only pro-inflammatory cytokines such as IL-1β, IL-18, IFN-γ, IL-6, (Fig. 1C), and TNF-α, IL-1α and LIF (Figs S3a and S4c) but also anti-inflammatory cytokines such as IL-4, IL-10 (Fig. 1D) and IL-13 (Fig. S3d) were increased in the peripheral blood, in comparison with mock-immunized mice. In ApoE^−/−^ mice, no inflammatory genes were modified in the liver following ompK36 immunization, and only Interferon Receptor Substrate-1 (IRS-1) expression was significantly reduced (−1,67) (Fig. 2B). Conversely, several significant changes were found in the visceral adipose tissue: anti-inflammatory genes such as IκBα (1,39), RORC (RORγ) (1,76; p = 0.056) were increased and pro-inflammatory cytokine genes such as IL-6 were decreased (−2,27) (Fig. 2D). In addition, JNK1 (MAPK8) (1,21), IL-1 receptor (IL1R) (1,52), Tyk2 (2,19), IFNαR1 (1,53) genes were increased in the adipose tissue of ompK36-immunized ApoE^−/−^ mice with respect to mock-immunized ones (Fig. 2D), suggesting an immune-metabolic activation. As observed in WT mice, no significant differences in the gene expression profiles of the spleen, PBMCs, or striated muscles were observed as a result of immunization.

Interestingly, these changes were observed several months after the last burst of immunization, supporting the hypothesis of a long-lasting adaptation of the immune-metabolism.

### Changes in microbiota composition are modulated by genetics and immunization

**Figure 3:**
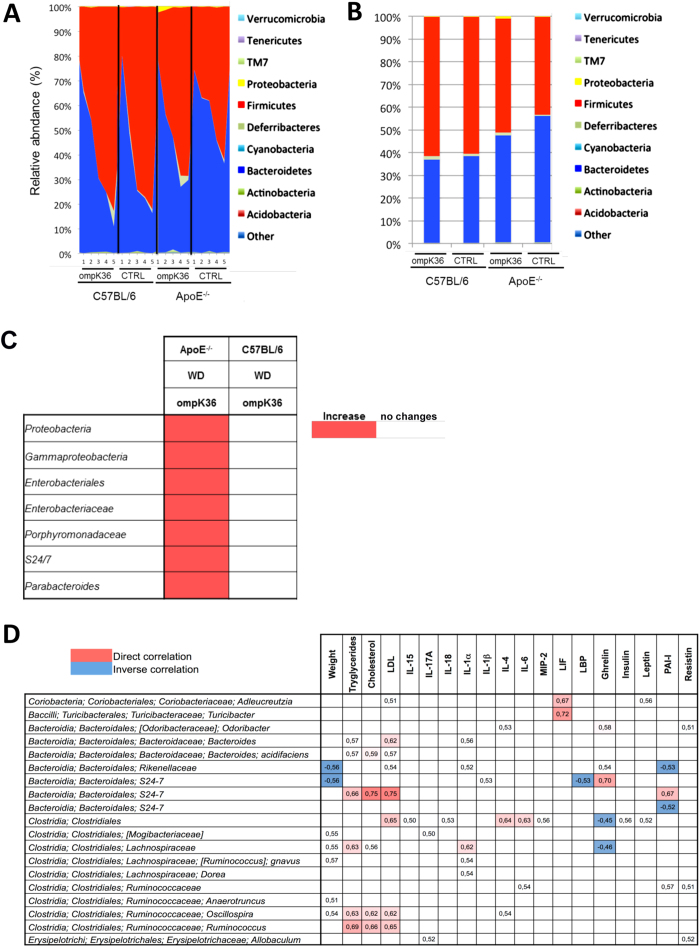
Composition of the colon microbiome in ompK36-immunized and mock-immunized mice. (**A**) Area chart of ompK36 immunized and control mice. (**B**) Bar chart of the average group composition (n = 7/group). (**C**) Bacterial clade was significantly (P < 0.05) influenced by ompK36 immunization compared to paired controls in ApoE^−/−^ and WT mice. Data are plotted as mean ± s.e.m. Statistical analysis: (one-way ANOVA with Bonferroni’s post hoc test and) student t test. (**D**) Heatmap graph of correlation analysis between bacterial OTUs and animal traits. Only statistically significant correlations are reported (r factor is shown). Analysis of all mice (n = 28) was performed by QIIME software.

In agreement with previous literature^8^, in both strains of mice a high *Firmicutes/Bacteroidetes* ratio was observed (Fig. S5a). However, immunization with ompK36 significantly altered the composition of the GM only in ApoE^−/−^ mice (Fig. 3A–C). ApoE^−/−^ mice immunized with ompK36 exhibited a significant alteration in the proportions of some bacterial clades: *Bacteroides spp.* were decreased, whereas *S24-7, Proteobacteria and Porphiromonadaceae* were increased (Fig. S5b).

In both C57BL/6 and ApoE^−/−^ mice, the presence of specific Operational Taxonomic Units (OTUs), i.e specific bacterial strains, was significantly correlated with animal traits including weight, serum biochemical and inflammatory parameters and hormone levels (Fig. 3D).

### Gut and atherosclerotic plaques of immunized mice show reduced inflammatory cells and an increased M2 macrophage fraction

**Figure 4:**
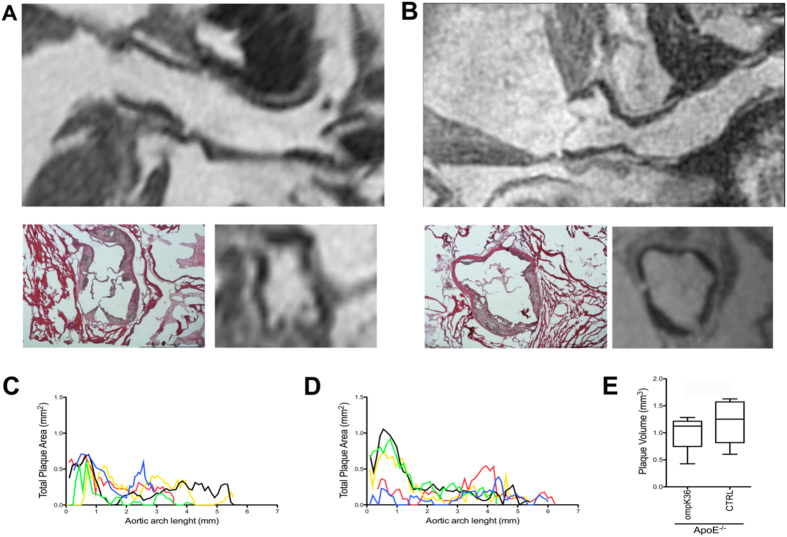
MRI evaluation of atherosclerotic plaques in ApoE^−/−^ mice. Representative longitudinal MRI images of the aortic root: (**A**) ompK36-immunized and (**B**) mock-immunized ApoE^−/−^ mice are shown in the upper row. The transversal MRI images of aortic plaques at sinus level are displayed in comparison with hematoxylin/eosin microphotographs (A, B lower row). Total plaque area distribution along the aortic root in 5 ompK36-immunized mice (**C**) and in 5 mock-immunized mice (**D**) is plotted. (**E**) The total plaque volume on the aortic root was calculated as Area Under Curve (AUC) and compared between the two groups. Data are presented as mean ± s.e.m. Statistical analysis: unpaired student t test (n = 5/group).

**Figure 5:**
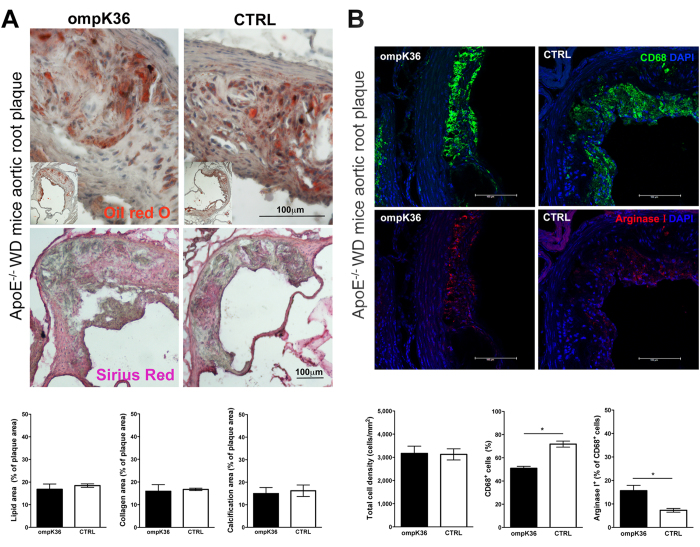
Morphologic characterization of aortic plaques at valvular inset level in ompK36-immunized ApoE^−/−^ and control mice. (**A**) Representative Oil red O (original magnification X40) and Sirius red (original magnification X10) images of aortic atherosclerotic plaques and quantification of the average lipid, collagen and calcification content by morphometry. Lipid, collagen and calcification areas were measured in at least five sections/mouse in 5 mice/group and were normalized on the total plaque area of each section. (**B**) Representative fields of aortic root sections stained with either anti-CD68 (green) or anti-Arginase I (red) (original magnification X40); quantification of total cell density, CD68^+^ cells, and ArgI^+^ cells are shown in the below histograms. Positive cells were counted in at least two sections/mouse and in at least 5 mice/group. Total plaque cells/plaque area were assessed and the percentage of CD68^+^ cells/total cell number calculated, while percentage of ArgI^+^ cells was calculated out of CD68^+^ cell number. Data are presented as mean ± s.e.m. (n = 5/group). Statistical analysis: unpaired student t test. *P < 0.05.

Atherosclerotic plaques of murine aortas were quantitatively analyzed by applying a novel *ex vivo* quantitative Magnetic Resonance Imaging (MRI) technique, specifically developed for this study. Plaques were successively analyzed by morphometry with transmitted light and fluorescence microscopy. In the aortic arches of ApoE^−/−^ mice, major atherosclerotic plaques were localized in the proximity of the aortic sinus, but no significant differences in plaque distribution along the aortic arch were demonstrated between ompK36-immunized and mock-immunized mice (Fig. 4A–C). MRI evaluated the volume and the number of plaques on the aortic root (as by morphometry) and along the entire length of the aortic arch through a fine-tuned operator-independent assessment. By MRI, the total volume of the aortic plaques in ompK36-immunized mice (mean value  =  1.1 mm^3^) showed a tendency to be lower than in mock-immunized mice however, statistical significance was not reached (Fig. 4E). Consistently, morphometry on Sirius red and Oil Red O-stained pseudo-serial sections of the aortic root did not reveal any significant difference among ApoE^−/−^ mice groups neither in plaque area, calcification, collagen, nor in lipid content (Fig. 5A). However, in the absence of significant differences in the total cell density of the aortic plaques, the percentage and density of CD68^+^ cells was significantly lower in ompK36 immunized ApoE^−/−^ mice with respect to mock-immunized mice. Along with the decrease in CD68^+^ cells, a significantly increased proportion of CD68^+^/Arginase I^+^ cells, suggestive of alternatively activated (M2) macrophages, was found in plaques of ompK36-immunized mice with respect to mock-immunized mice (Fig. 5B).

**Figure 6:**
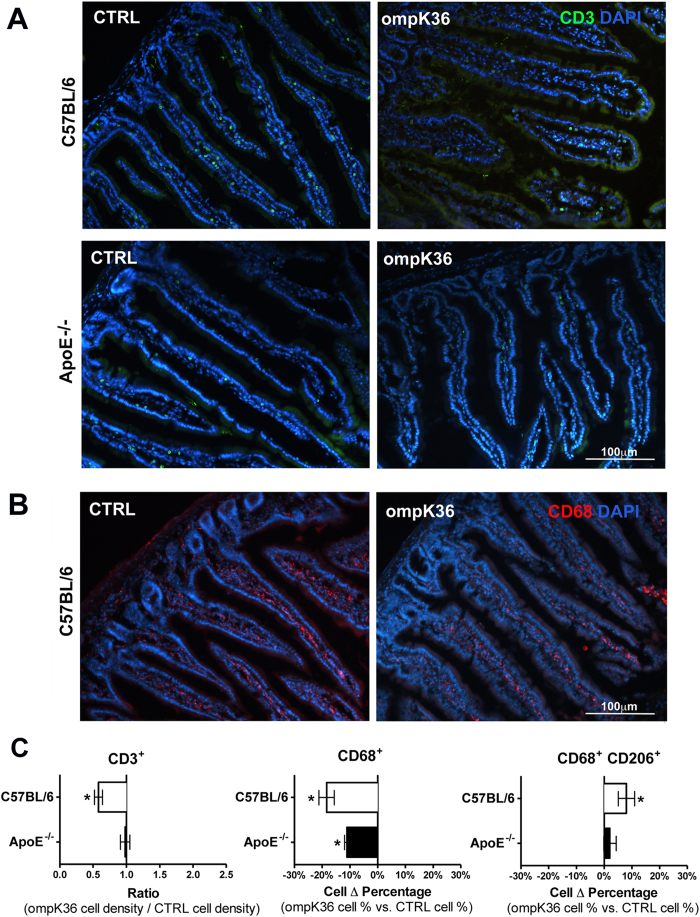
CD3^+^ and CD68^+^ cell localization in sections of mouse small intestines. Representative fields displayed (**A**) CD3 (green) or (**B**) CD68 (red) immunofluorescence (original magnification X20). (**C**) Quantitative analysis of CD3^+^, CD68^+^ and CD68^+^/CD206^+^ cell content in C57BL/6J and ApoE^−/−^ either ompK36-immunized or non-immunized control mice,is shown. CD3^+^ cell number was normalized on total villi area. CD68^+^ cells were normalized versus total cell number present inside villi (epithelial cells excluded). CD68^+^/CD206^+^ cells were normalized versus CD68^+^ cells. Data are presented as mean ± s.e.m. Cells were counted in at least 5 sections/mouse in 5 mice/group. Statistical analysis: unpaired student t test. *P < 0.05.

CD3^+^ and CD68^+^ cells were evaluated on sections of the small intestine (Fig. 6A,B). Quantitative analysis in C57BL/6 mice immunized with ompK36 demonstrated a lower density of CD3^+^ cells compared to mock-immunized mice (Fig. 6C). In ApoE^−/−^ mice, no difference was detected in immunized mice. In all mice, the percentage of CD68^+^ cells was lower in ompK36-immunized mice compared to mock-immunized mice, independently of apoE presence or deficiency. This suggests that apoE function is needed to amplify the immune modulating effects of immunization. Moreover, all of the ompK36–immunized C57BL/6 mice showed enrichment in CD68^+^/CD206^+^ alternatively activated macrophages compared to mock-immunized mice; while ApoE^−/−^ mice failed to show significant differences in the number of CD68^+^/CD206^+^ cells on gut sections (Fig. 6C).

## Discussion

We have demonstrated that adaptive responses following active immunization against *Enterobacteriaceae*, enhanced local and systemic immune-control by eliciting and maintaining a new equilibrium with bacteria present in the gut. In these settings, the presence/deficiency of apoE, fundamental for the proper activity of the machinery, has physiological consequences on the host’s metabolic and inflammatory status.

In fact, the enhanced circulating levels of apoE observed in immunized C57BL/6 animals could promote Toll-like Receptor (TLR) 3 and 4 inhibition, macrophage differentiation^9^, LPS hepatic clearance^10^, and can attenuate inflammatory responses by binding to LPS^11^. A direct physiological link between apoE immunomodulation and adaptive immune response against bacterial OMPs or its outcome was still unknown. ApoE, produced and released by many cell types (hepatocytes, smooth muscle cells, neuronal cells) represents up to 10% of proteins constitutively secreted by macrophages^12^ after contact with immune complexes and Fcγ receptor engagement^13^. In this study, we demonstrate that, when “guided” by the adaptive immune response, apoE plays a key role in tuning a local host immune response, acting on sites where frequent and elevated inflammatory reactivity could be detrimental. In conditions of low-grade chronic inflammation triggered by WD, previously described as metabolic endotoxemia or “metaflammation”, pro-inflammatory cytokines increase in the serum and influence the development of metabolic diseases, such as insulin resistance, metabolic syndrome, atherosclerosis and NAFLD/NASH^14^,^15^,^16^,^17^. Interestingly, IL-1β, IL-6, IFN-γ, CXCL2 (MIP-2), IL-18, IL-15, fasting glucose and insulin levels were significantly reduced in C57BL/6 mice upon immunization against ompK36, suggesting that eliciting a long lasting systemic immune response against antigens of GM microbes can confer significant protection on the murine WD-associated host dysmetabolism, similar to that observed in human metabolic diseases.

The key role of apoE in this protective mechanism was evident when immunization was performed on ApoE^−/−^ animals. In ApoE^−/−^ mice, gut immunomodulation was not as effective as in C57BL/6 wild type animals, since the composition of the GM and the circulating cytokines were highly susceptible to perturbations upon immunization. However, the expression of pro-inflammatory cytokines within visceral adipose tissue was decreased and a tendency to develop smaller atherosclerotic lesions was also present. These effects may be due to the decreased total number of macrophages and to the increased proportion of alternatively activated macrophages (M2), which have been related in humans to stable asymptomatic plaques^18^. Although the increase in circulating cytokines observed in ApoE^−/−^ mice cannot be associated to a specific pattern, it resembled, with a reduced intensity, the “cytokine storm” observed in gram-negative sepsis or LPS-induced shock^19^. Fcγ receptor engagement, alone, triggers cytokine production by macrophages in response to LPS *in vitro*^20^. Therefore, immunization of C57BL/6 wild type mice with ompK36 allows simultaneous FcγR and TLR engagement and *in vivo* apoE release. This can explain the enhanced commitment of macrophages toward an anti-inflammatory phenotype, the lower number of CD3^+^ T lymphocytes and macrophages in the gut^21^,^22^,^23^,^24^, as well as the considerably reduced levels of circulating cytokines observed only in wild type mice.

Of note, in C57BL/6 mice, immunization decreased serum levels of the inflammasome-related IL-1β^25^,^26^,^27^. Interestingly, inflammasome-related inflammation has been suggested as risk factor for autoimmune and immuno-metabolic diseases including metabolic syndrome and and NAFLD^28^,^29^, and frequently associated to intestinal infections and reactions to dietary antigens potentially capable of altering normal intestinal function. Moreover both IL-6 and the expression of its receptor (gp130) were recently demonstrated to affect macrophage infiltration during atherosclerosis in ApoE^−/−^ mice^30^ and the risk of myocardial infarction in humans^31^.

Importantly, the role of apoE immunomodulatory functions in the context of both adaptive and innate responses against other microbes or against self-antigens such as oxidized-LDL should also be taken into consideration. Interestingly, in fact, Binder’s group a few years ago showed that pneumococcal vaccination decreases atherosclerotic lesion formation through a molecular mimicry mechanism between *Streptococcus pneumoniae* and oxidized LDL^32^. It was later demonstrated that natural immunity against the cross-reactive phosphorylcholine epitope or against modified LDL was associated with protection from atherosclerosis^33^,^34^,^35^,^36^,^37^. In contrast, other groups suggested that adaptive immune responses contribute to the development of atherosclerosis by promoting inflammation and plaque growth^38^,^39^,^40^. However, the roles of both innate and adaptive immune responses were mostly investigated using the ApoE^−/−^ mouse model of atherosclerosis and our results may help to clarify the apparently contrasting results observed. Future studies should in fact clarify to what extent apoE function affects innate or adaptive responses against oxidized-LDL or exogenous antigens in animals or in humans, since its systemic immunomodulatory functions may affect both circulating cytokine composition or local macrophage differentiation that at least in part, mediate both the oxidation of LDL and the inflammatory-cell composition of the plaques.

In our settings, apoE appeared also to be a key regulator in the homeostasis of gut immunity, whose pleiotropic functions strongly influenced the blood and intestinal microenvironment. Consistently, in C57BL/6 animals, systemic immunization did not affected GM composition, including the proportion of *Enterobacteriaceae*; whereas in ApoE^−/−^ mice, immunization contributed to modify GM diversity and composition. In ApoE^−/−^ mice, after immunization with ompK36 less *Bacteroides* but increased *Proteobacteria*, *S24-7*, *Porphyromonadaceae* and *Enterobacteriaceae* were found with patterns similar to those observed in inflammatory conditions as diabetes, metabolic syndrome and NAFLD^41^,^42^,. This suggests that both local and systemic responses against bacteria present in the gut are strongly influenced by apoE function and strongly shape the composition of GM. Alteration in GM composition and function has been associated with human disorders such as metabolic syndrome, diabetes, obesity^14^,^43^,^44^ and atherosclerosis, including its acute complications, i.e. stroke or myocardial infarction^45^,^46^,^47^. However, the environmental or genetic factors responsible also for affecting GM diversity and accounting for the multifactorial pathogenesis of these diseases require integrated “omics” approaches. In mice, a hyperlipidic diet per se was shown to modify GM composition, increase gut permeability, and alter the systemic inflammatory profile^17^,^48^.

*Enterobacteriaceae*, present in the GM of healthy subjects, can cause opportunistic infections only in situations of altered immunity or gut barrier function. It cannot be excluded that immunization with antigens of other members of the GM has similar effects. However, it can be speculated that the different *in vivo* exposure to gut bacteria, the different ecological niches present in the gut, or the breadth of the immune response against bacteria with homologous or cross-antigenic OMPs may affect the biological consequences of immunization. Nasal immunization with the OMP of P*orphyromonas gingivalis* was previously shown to be protective against accelerated atherosclerosis induced by intraperitoneal inoculation of bacteria in ApoE^−/−^ mice. In this paper we demonstrate the effect of immunization in wild type and in ApoE^−/−^ animals after almost five months from last immunization, and in the absence of bacterial challenge. *P. gingivalis* mainly colonize only the oral cavity, while *Enterobacteriaceae*, such as *K. pneumoniae* or *P. mirabilis* (the antibodies that we previously cloned from human plaques and those elicited in these animals, react against several cross-antigenic OMPs, including OmpK36, OmpK35 and OmpF from other *Enterobacteria*; data not shown), can adhere to the gut mucosa both in the small and in the large intestine, transiently translocating through the epithelial barrier, a process facilitated by WD, thus constantly in challenge with the gut immune system^49^,^50^. Moreover, gram-negative bacteria such as *Enterobacteriaceae* can release Outer Membrane Vesicles (OMVs) made up of the outer membrane components containing LPS, OMPs, other virulence factors and Pathogen Associated Molecular Patterns. OMVs can be detected in human tissues and can trigger local and systemic inflammatory responses^51^,^52^,^53^. Through the circulation, bacteria can reach visceral fat or atheromas^2^, directly promoting local inflammation or eliciting specific immune responses^54^,^55^, and indirectly influencing host metabolism and systemic inflammation^56^,^57^.

The lifestyle in developed countries has probably decreased the chances of building a proper immune equilibrium early in life with members of the GM, such as *Enterobacteriaceae* or with pathogens that can be frequently encountered during life in developing countries. Modified habits in Western lifestyles have led to an increasing number of inflammatory and autoimmune diseases, as suggested several decades ago by the supposed “Hygiene Hypothesis” that explains this fact by an increase on hygiene standards and lack of infectious stimuli during the early postnatal period^58^. Consequently, innate modulators of local and peripheral immune activation such as apoE cannot be physiologically exploited. It was also suggested that the composition of intestinal microbiota plays a key role in the development of inflammatory diseases by influencing host immune responses and the development of regulatory T cells^59^. Recently, Stepankova *et al*.^46^ have shown that ApoE^−/−^ mice bred under germ-free conditions and fed with low cholesterol diet develop atherosclerotic lesions that do not develop in conventionally reared mice fed with the same diet. Thus suggesting that intestinal microbiota have a crucial protective effect against the formation of atherosclerotic plaques, that as we show in this paper may be, at least in part, mediated by the adaptive immune response against gut microbes.

In conclusion, Active immunization against *Enterobacteriaceae* can persistently restore the equilibrium between the host and the GM, by triggering apoE-mediated immune regulation and leading to a reduced systemic inflammatory steady state. However, in humans, ApoE exists in 3 common isoforms and numerous reports have established that only *APOE4* allele is associated with an increased risk of premature atherosclerosis^7^. Similarly, its immunomodulatory properties were shown to be isoform-dependent^4^,^5^ independently of its role in lipoprotein metabolism^6^. Thus we cannot exclude that the observed mechanism could have different effects in humans bearing distinct ApoE alleles. However, future active or passive immunotherapeutic approaches for immune-metabolic diseases and to protect from atherosclerosis progression or degeneration, should be considered in humans by selectively targeting members of the GM.

## Materials and Methods

### Preparation of bacterial antigens

OmpK36 gene was amplified from *Klebsiella pneumoniae* lysate by using specific primers and cloned into a pET-28b expression vector (Novagen). Induction of protein expression was performed following manufacturer instructions (pET System Manual - Novagen), using a final IPTG concentration of 1 mM. Immobilized Metal Affinity Chromatography (IMAC) purification of ompK36 was performed following manufacturer instructions (QiaExpressionist - Qiagen) using *E. coli* BL21(DE3) bacterial cells and Ni-NTA resin (QIAGEN).

### Ethical Statement

All experimental procedures were reviewed and approved by the Institutional Animal Care and Use Committee (IACUC) at the Department of Veterinary Science of Università degli Studi di Milano (2011-Nr 3) and follow the European regulations for animal experimentation (Directive 2010/63/EU). All the methods were carried out in accordance with the approved guidelines.

### Animals and diet

Forty- 6-week-old male ApoE^−/−^ and C57BL/6 mice (Charles River Laboratories) were single-housed in Specific Pathogen-Free (SPF) conditions with free access to food and water. A red tunnel (VWR International) was placed in every cage for environmental enrichment. After weaning, mice were fed with Normal Diet (ND) containing 3% (w/w) fat (4RF21 certificate - Mucedola). At 6 weeks of age they were switched to a Western Diet (WD) consisting of 21% (w/w) fat and 0,2% cholesterol (TD88137 – Harlan) for an additional 24 weeks.

### Experimental plan

At 6 weeks of age, mice were randomly assigned to different groups and immunized. (Fig. S1a) For the first immunization the antigen, i.e. ompK36, or adjuvant only (recombinant mouse IL-12, R&D systems, 1 μg IL-12 in 150 μl of sterile Dulbecco’s PBS), was administered intra-peritoneally (50 μg/antigen in adjuvant solution). In successive bursts (8, 10 and 12 weeks of age) it was injected subcutaneously (25 μg per antigen in 150 μl in adjuvant solution) (Fig. S1b). Mouse weight and the amount of consumed chow were monitored every week until sacrifice. At 30 weeks of age, mice were anesthetized by isofluorane inhalation and blood samples collected in sodium-EDTA, centrifuged (3000 rpm for 15 min) and stored in aliquots at −80 °C. Mice were sacrificed by excess of anesthesia, then infused with gadolinium-based contrast agent (Magnevist, Bayer) for MRI via left ventricle, followed by PBS and 4% paraformaldehyde in PBS. Specimens (heart with aorta, small intestine) were fixed overnight and cryoprotected in sucrose 10%, then embedded in OCT compound (Killik, BioOptica), frozen in isopentane/liquid nitrogen and stored at −80 °C. Liver, visceral adipose tissue, spleen, PBMC, large intestine, striated muscles and fecal material were harvested in RNAlater (Qiagen) and kept at −80 °C until usage.

### Serum titers against bacterial antigens

ELISA assays were performed as previously described (27). Briefly, 1 μg per well of purified ompK36 was coated in PBS1x pH = 7,4 at 4 °C overnight in a microtiter plate (Corning). Plates were blocked with PBS/1% BSA for 1 h at 37 °C. Four washes were performed in an automated washer (Thermo Scientific) with PBS/0.05% Tween-20. Plates were then blocked with PBS/3% Milk/0.02% Sodium Azide for 2 h at 37 °C. Mouse sera diluted in PBS/1% BSA were incubated for 1 h at 37 °C. After washing 5 times with PBS/0,1% Tween-20, Goat anti-Mouse IgG (Fc specific) antibody (Sigma) (1:10000 in PBS/1% BSA) was incubated at 37 °C for 1 h. After washing with PBS/0,1% Tween-20, TMB solution (Pierce) was added in every well and incubated at 37 °C for 15 min. The colorimetric reaction was stopped with H_2_SO_4_ 1 M solution and the Optical Density (O.D.) was measured at 450 nm by MultiSkan GO microplate reader (Thermo Scientific). Serum antibody titer was defined as the reciprocal of the dilution with mean O.D. two times higher than background. Only blank and secondary antibodies were included in every ELISA assay as controls.

### Plasma analyses

Total cholesterol, triglycerides and LDL were evaluated at sacrifice by Ospedale San Raffaele Mouse Clinic (Milan - Italy). Circulating cytokines, chemokines and hormones were measured on stored samples by using Bio-Plex assays (Pro Mouse Diabetes Assay, Mouse Group I: 23-plex panel, Mouse Group II: 9-plex panel and Mouse Adiponectin assay) following manufacturer’s instruction using Bio-Plex 200 Suspension Array System (Biorad). Circulating apoE protein was measured by ELISA assays (mouse apoE Elisa Kit (USCN Life Science Inc.)).

### Gene expression

Total RNA was extracted from homogenized tissues using RNeasy Mini kits (Qiagen). RNA was reverse-transcribed using RT2 First Strand Kit (Qiagen). One microgram of purified RNA was analyzed in every plate using Mouse Fatty Liver (PAMM-157Z, Qiagen) or Mouse Innate and Adaptive Immunity (PAMM-052Z, Qiagen) arrays. Reactions were performed using the RT2 SYBR Green/ROX Mastermix (Qiagen) and AbiPrism 7900HT Fast Real-Time PCR System (Life Technologies) following manufacturer’s instructions. Six different House-Keeping Genes (HKGs) were evaluated in every tissue: beta-actin (Actb), beta-2-microgobulin (B2m), glyceraldehyde 3-phosphate dehydrogenase (Gapdh), beta glucoronidase (Gusb), heat shock protein 90 kDa alpha class B member 1 (Hsp90ab1) and ribosomal protein L32 (Rpl32). Post-amplification melting curve analysis was performed to check for unspecific products and RT, PCR and genomic DNA controls were included to ensure the absence of inhibitors and genomic DNA contaminant. Threshold cycles (Ct-values) were normalized to at least 2 HKGs used within each sample to obtain sample-specific ΔCt values (=Ct gene of interest − Ct HKG). 2−ΔΔCt values were calculated to obtain fold expression levels, where ΔΔCt  =  (ΔCt treatment − ΔCt control). Analyses were performed with web-based Sabiosciences software available at http://pcrdataanalysis.sabiosciences.com/pcr/arrayanalysis.php (v3.5) and differences were considered significant if P < 0.05.

### Microbiome analysis

Bacterial DNA from 50 mg of fecal material and the surrounding colonic mucosa was extracted using PowerFecal DNA Isolation Kit (MoBio), following manufacturer’s instruction, but lysis time was increased to 15 min. Purified DNA was quantified and 200 ng per reaction was used to amplify 16S V3–5 regions using barcoded sample-specific primers, AccuPrime Taq Polimerase (Invitrogen) and the following cycling protocol: 95 °C for 5 min, 40 cycles of (95 °C for 30″, 55 °C for 45″ and 72 °C for 1 min) and stored at 4 °C until usage. Amplicons were loaded on 1% agarose gel and purified with QiaQuick Gel Extraction kit (Qiagen). Extracted amplicons were purified with AMPure XP beads (Beckman Coulter) and used for emulsion-PCR and ultra-deep pyrosequencing following 454 GS Junior manufacturer’s instruction (Roche). After quality filtering, resulting sequences (>250bp) were analyzed with QIIME software (1.6.0). The GM was analyzed in fecal samples from 7 mice/group with at least 15000 high-quality sequences/sample. Good’s coverage index was >0,99 for all the samples. Correlation between plasma proteins levels and single OTU were evaluated in QIIME with Bonferroni’s correction.

### Magnetic Resonance Imaging (MRI) of atherosclerotic plaques

OCT-embedded heart and aorta were melted and washed with PBS1X and then included in 20% porcine gelatin (Sigma) in PBS 1% Pen./Strep. to insure stability in *ex vivo* MRI procedures. MRI was performed by a 7 Tesla small-bore scanner (Bruker BioSpec 70/30) equipped with a circular polarized body volume coil. MRI protocol included a 3-D RARE T2-weighted sequence (TR  =  2800 ms; TE  =  40 ms; NEX  =  6) with isotropic voxel size. The acquired datasets were transferred on 32-bit OsiriX software (v7.7.1) for post-processing using curved multiplanar reformation (MPR) method. Both straightened/stretched long-axis views and short-axis trans-axial views of the aorta were obtained. Different frames of continuous short-axis images with a mean thickness of 100 μm were exported for each atherosclerotic plaque detected on long-axis curved MPR views of the aorta. Using these new datasets of short-axis views of the aorta, the areas of the aortic lumen and the atherosclerotic plaque were measured and summed up to obtain a volume on ImageJ 64-bit (v1.47). The aortic arch, in which plaques were evaluated more deeply, were defined from the beginning of the aortic root to the subclavian artery. The plaque volumes and plaque distribution along aortic arches were calculated from the obtained data.

### Histology and morphometry

Serial cryosections from the aortic root (10-micron thick) were obtained from 5 mice/group and were destined to either Hematoxylin/Eosin or Oil Red O or Sirius Red staining, following standard methods. Either lipids, collagen, or calcifications were measured in the aortic root cryosections. Images of aortic root sections were taken using the Eclipse55i microscope equipped with a DS-L1 camera and Lucia G software (all from Nikon, Tokyo, Japan). At least 6 sections/mice distanced 300 μm apart were obtained and analyzed by using ImageJ software. Either the total plaque areas, or the areas occupied by lipid, collagen and calcifications were selected as Region Of Interest (ROI). After conversion to binary, a suitable threshold was selected and the areas measured. For all the considered parameters, the percentage of areas/section was calculated and all the values/mouse/group were averaged. Reagents were purchased from BioOptica and Sigma.

### Immunofluorescence and morphometry

Cryosections (10 microns thick) of the aortic root and arch, and of the small intestine were tested by immunofluorescence for the presence of macrophages and T-Lymphocytes. Sections of the aorta and/or small intestine were rinsed in Dulbecco’s PBS, permeabilized with 0,5% Triton-X100 in PBS (1 h room temperature, RT). Aspecific binding on aortic tissue was blocked by 1% BSA in PBS (1 h, RT), in the small intestine by 10% Donkey serum (Sigma-Aldrich)^60^. The sections were incubated with opportune primary antibodies. Goat anti-ArgI (sc-18351, Santa Cruz Biotechnology, CA, diluted 1:50, 4 °C overnight), Rat anti-Mouse CD68 (clone FA-11, BioLegend, diluted 1:100, 2 h RT), Goat anti-Mouse CD206 (sc-34577, Santa Cruz Biotechology, diluted 1:50 in 5% Donkey serum, 0,3% Triton-X100 in PBS, 4 °C overnight), Rabbit anti-Mouse CD3 (ab5690, Abcam, diluted 1:50, 2 h RT), were used, revealed either by Donkey anti-Goat-IgG-AlexaFluor488 (A-11055) anti-Rabbit-IgG-AlexaFluor488 (A-21206) and Donkey anti-Rat-IgG-AlexaFluor594 (A-21209, Molecular Probes, Life Technologies Inc.) all diluted 1:500 and applied for 45 min RT. Nuclei were counterstained with 4,6-diaminidino-2-phenylindole (Sigma-Aldrich, 0.2 nmol/L, diluted 1:250, 10 min. RT). Finally, slides were mounted with FluorSave (Merck) and analyzed under an Eclipse55i fluorescence microscope equipped with a DS-L1 camera and Lucia G software (Nikon, Tokyo, Japan). Cell counts were performed using ImageJ software. In the aortic root at least 400 cells/section in at least two sections/mouse in 5 mice/group were counted. Total cell density was calculated, the proportion of CD68^+^ cells was obtained as a the ratio CD68^+^ cells/total cell number; the percentage of ArgI^+^ cells was calculated as percentage of the ratio ArgI^+^ cells/CD68^+^ cell number. In sections from the small intestine, a minimum of 100 cells/section was counted in at least 5 sections/mouse in 5 mice/group. The CD3^+^ cell density was calculated as the ratio of CD3^+^ cells/total area occupied by intestinal villi (determined similarly to plaque area by ImageJ software). CD68^+^ cells were determined by the percentage of CD68^+^ cells/total cell number inside villi (epithelial cells excluded). The percentage of CD68^+^/CD206^+^ cells was also calculated.

### Statistical analyses

All data sets were tested for normality with the D’Agostino-Pearson omnibus K2 normality test with a significance level set to p  =  0.05 before the appropriate parametric or nonparametric statistical comparison test was carried out using Graphpad Prism (v5.0 – GraphPad Software) and JMP (v11 - SAS) software. Data were plotted as the mean ± Standard Error of the Mean (S.E.M.). Differences between two groups were assessed using a two-tailed, unpaired Student t test with Welch’s correction, if required. Considering the number of mice or samples analyzed, and the observed variability, the statistical power of the analyses was between 0,8 and 1,0 for all parameters. Significant differences are indicated in the figures by *p < 0.05, **p < 0.01 and ***p < 0.001.

## Additional Information

**How to cite this article**: Saita, D. *et al*. Adaptive immunity against gut microbiota enhances apoE-mediated immune regulation and reduces atherosclerosis and western-diet-related inflammation. *Sci. Rep.* **6**, 29353; doi: 10.1038/srep29353 (2016).

## Supplementary Material

Supplementary Information
