# Supplementary material for: Adaptive immunity against gut microbiota enhances apoE-mediated immune regulation and reduces atherosclerosis and western-diet-related inflammation
Source: Sci Rep. 2016 Jul 7;6:29353. doi: 10.1038/srep29353 (PMC4935993; doi:10.1038/srep29353)
Supplement: Supplementary Information [file srep29353-s1.pdf]

Adaptive immunity against gut microbiota enhances apoE-mediated immune regulation and reduces atherosclerosis and western-diet-related inflammation

Diego Saita[1], Roberto Ferrarese[1], Chiara Foglieni[2], Antonio Esposito[3,4], Tamara Canu[4], Laura Perani[4], Elisa Rita Ceresola[6], Laura Visconti[1], Roberto Burioni[1,5], Massimo Clementi[1,5] & Filippo Canducci[1,6]

[1] Microbiology and Virology Laboratory, San Raffaele Scientific Institute IRCCS, Milan, Italy.

[2] Cardiovascular Research Area, San Raffaele Scientific Institute IRCCS, Milan, Italy.

[3] Department of Radiology, San Raffaele Scientific Institute IRCCS, Milan, Italy.

[4] Centro Imaging Sperimentale (CIS), San Raffaele Scientific Institute IRCCS, Milan, Italy.

[5] Faculty of Medicine, Vita-Salute San Raffaele University, Milan, Italy.

[6] Department of Biotechnology and Life Sciences, Insubria University, Varese, Italy.

Correspondence should be addressed to F.C. (canducci.filippo@gmail.com).

SUPPLEMENTARY RESULTS

A

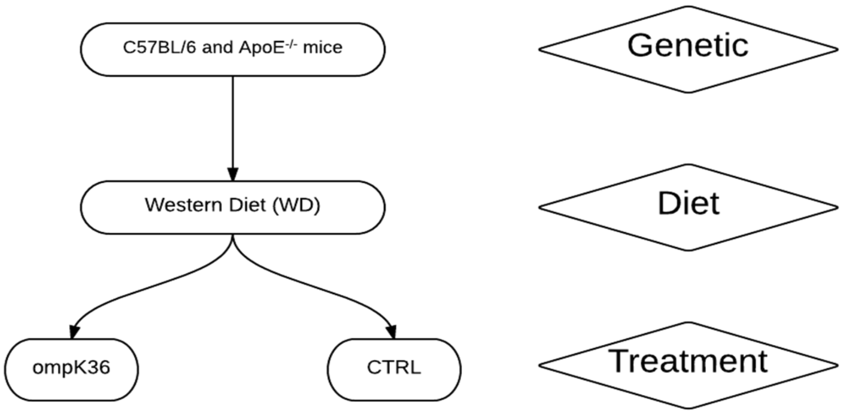

B

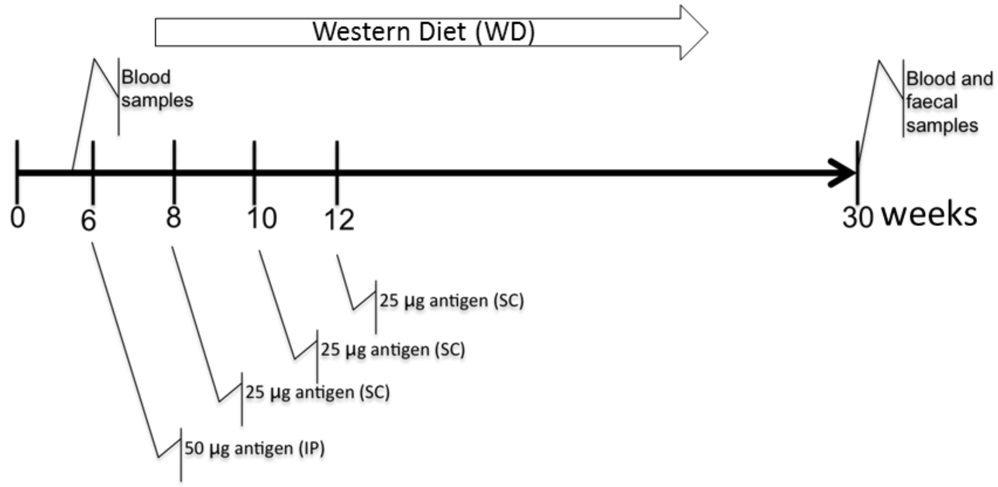

C

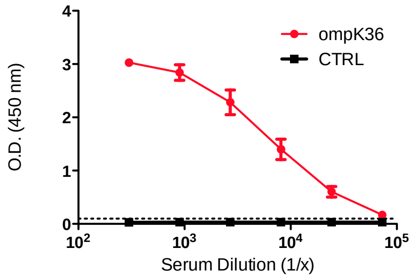

Supplementary Figure 1 Study design, immunization protocol and serum antigen-specific IgG antibody titer. C57BL/6 and ApoE$^{-/-}$ were immunized with a bacterial antigen and IgG serum titer was evaluated at sacrifice (nearly 18 weeks after last immunization) by ELISA assay (n=14/group). (A) Study design. (B) Immunization protocol, mIL-12 was used as adjuvant. SC: subcutaneous, IP: intra-peritoneal. (C) Serum antibody titer was defined as the reciprocal of the dilution with mean O.D. two times higher than background (dotted lines). Data are plotted as mean ± s.e.m.

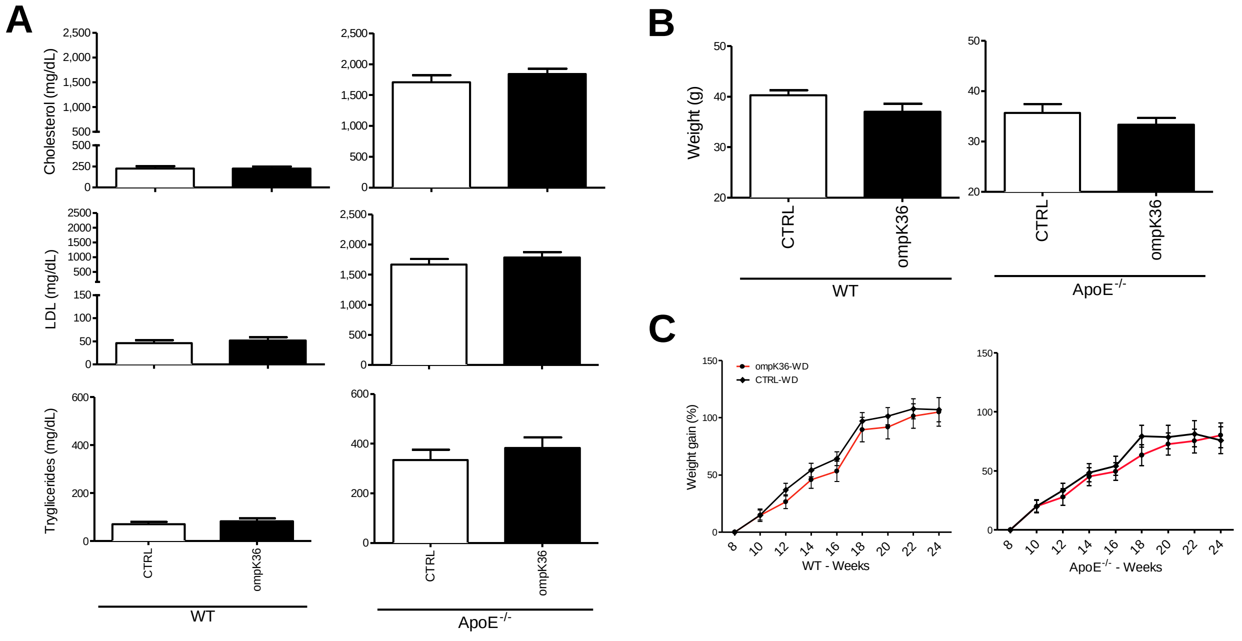

Supplementary Figure 2 Serum lipid profile, weight and glycemia. Mice were weighed every two weeks since weaving, while serum parameters were evaluated at sacrifice (n=7/group). (A) Serum lipid profile of ApoE$^{-/-}$ and WT mice. (B) Weight of mice at sacrifice. (C) Relative weight gain throughout experimental time course. Statistical analysis was performed by unpaired student t test (n=7). Data are plotted as mean ± s.e.m.

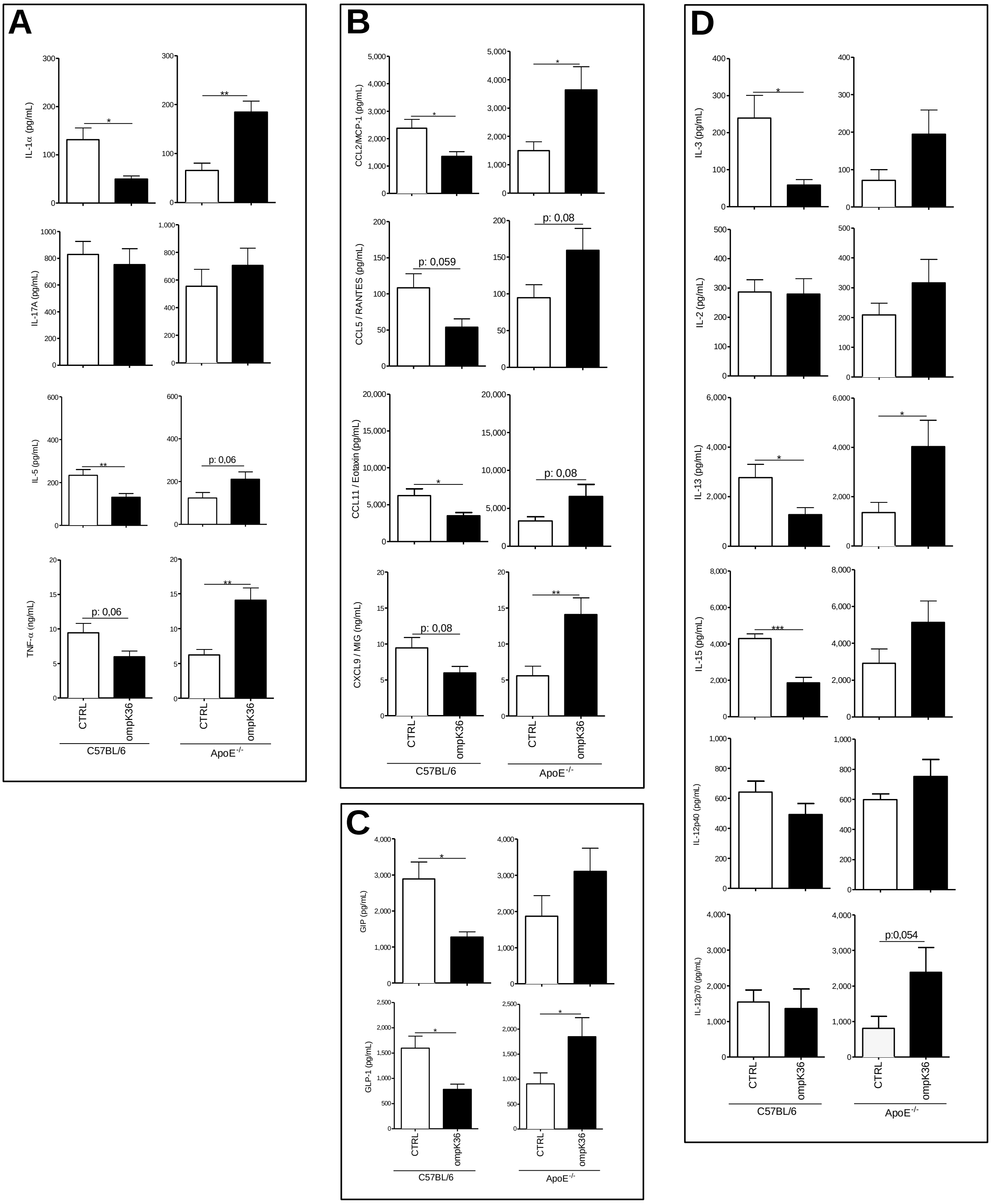

Supplementary Figure 3 Blood content of inflammatory mediators at the time of sacrifice. ApoE$^{-/-}$ and C57BL/6J mice fed with WD were analyzed separately. (A) pro-inflammatory cytokines, (B) chemokines, (C) incretins, and (D) other cytokines. (n=7/group). Unpaired student t test with Welch's correction, if needed, assessed the difference between groups. Data are plotted as mean ± s.e.m. *: P<0,05; **: P<0,01. Differences close to significance (0,05<P<0,10) are indicated in figures as exact P value.

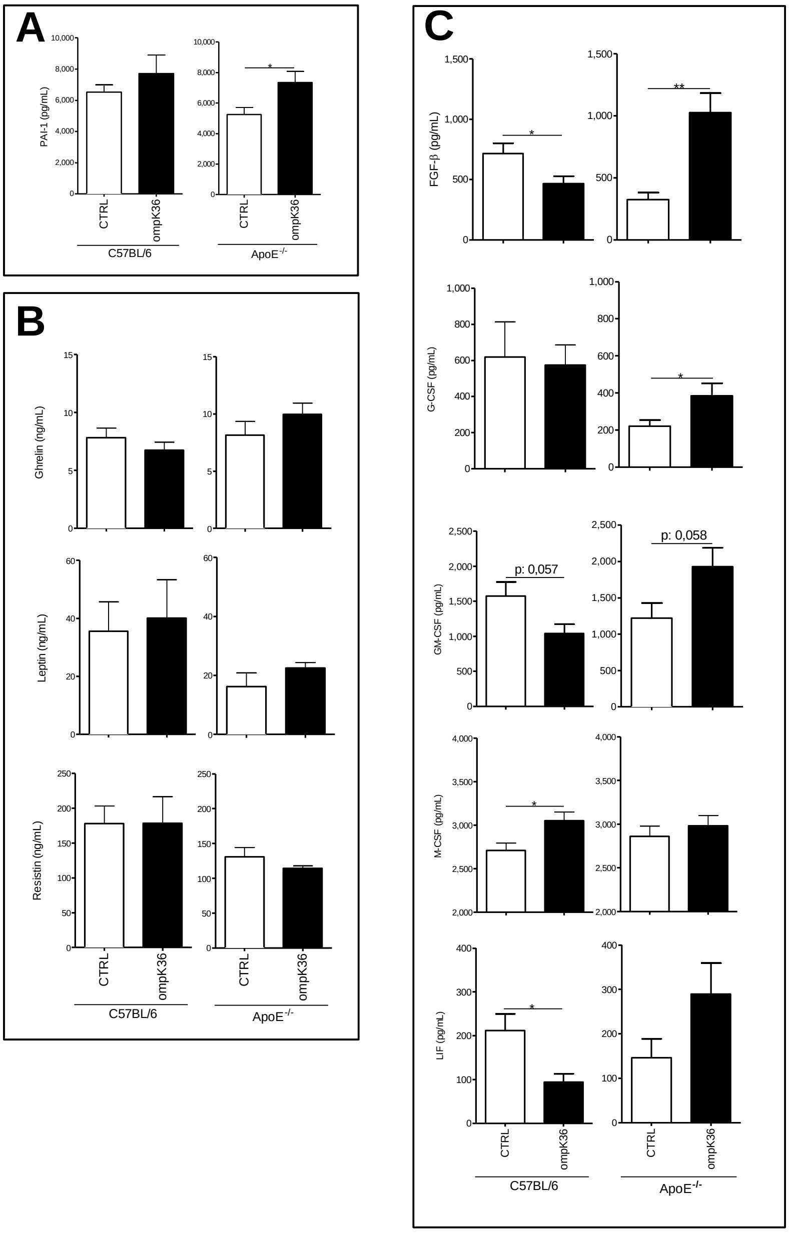

Supplementary Figure 4 Blood content of inflammatory mediators in all mice at the time of sacrifice. ApoE$^{-/-}$ and C57BL/6 mice fed with WD were analyzed separately. (A) PAI-1, (B) hormones and (C) growth factors. (n=7/group). Unpaired student t test with Welch's correction, if needed, assessed differences between groups. Data are plotted as mean ± s.e.m. *: P<0,05; ** P<0,01. Differences close to significance (0,05<P<0,10) are indicated in figures as exact p value.

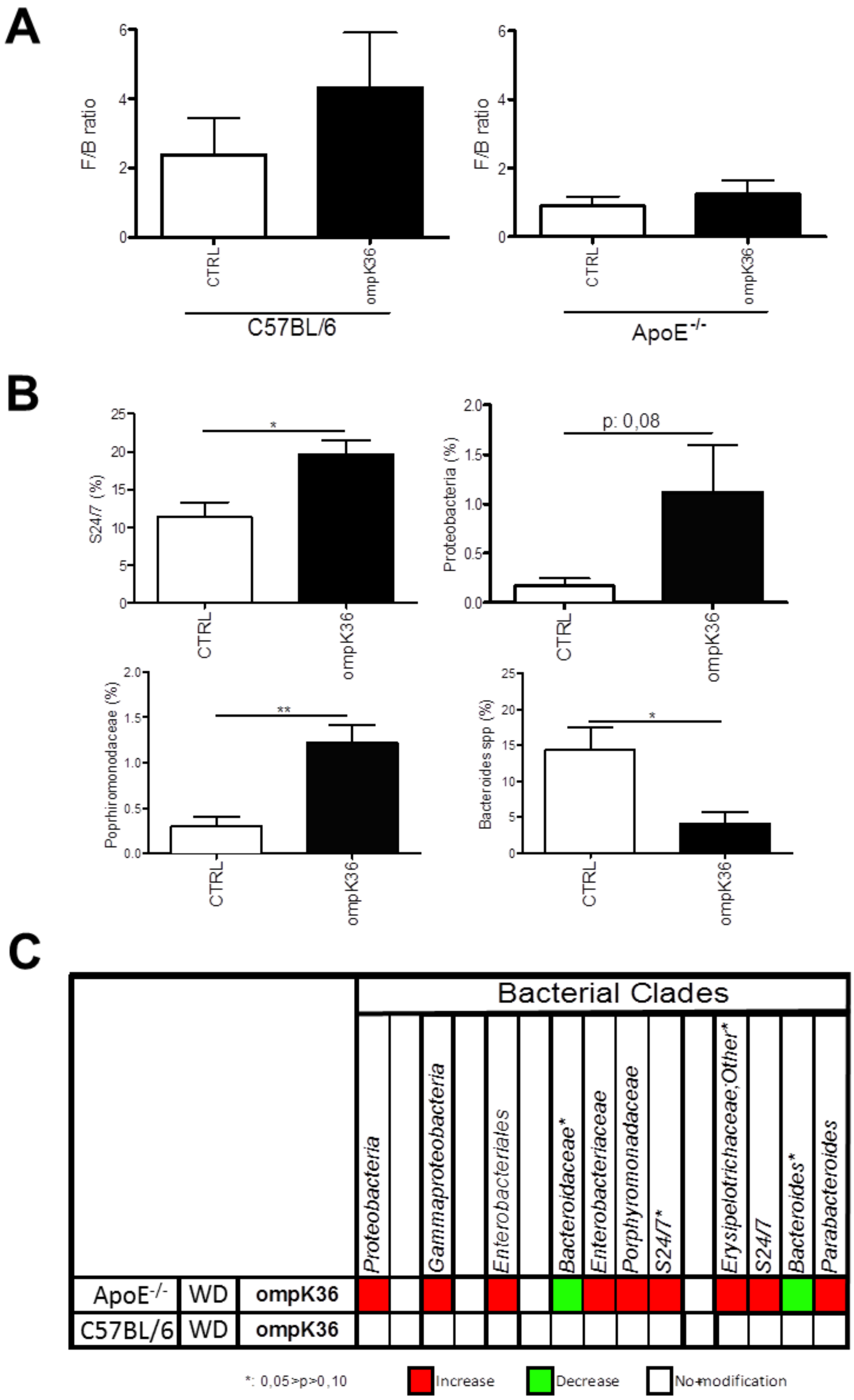

Supplementary Figure 5 Composition of the colon microbiome in all of the mouse groups. (a) Firmicutes-to-Bacteroidetes ratio in ApoE$^{-/-}$ and C57BL/6J mice. (b) Bacterial clades modified by ompK36 immunization compared to mock-immunization in ApoE$^{-/-}$ mice. (c) Bacterial clades significantly affected by immunization with different bacterial proteins compared to paired mock-immunized controls in ApoE$^{-/-}$ and C57BL/6 mice. Data are plotted as mean ± s.e.m. Statistical analysis: one-way ANOVA with Bonferroni's correction and student t test.
